# Institutional Housekeeping

**Published:** 1912-01-20

**Authors:** 


### INSTITUTIONAL HOUSEKEEPING.

(Workers' Questions and Criticism of every kind will be welcomed.)

### Questions and Answers on Practical Points.

"ORPHANAGE" asks for a good tasty method of preparing haricot beans for children who do not like curry.

Soak half a pint of the beans in cold water over night. Care should be taken in purchasing the beans to see that they have not been in stock a long time, as in that case they will always remain hard. They are at their best in autumn and winter, after the new crop comes in. Put the beans in a pan with cold water to cover them, and simmer them gently for two hours or until soft, the time depending on the age of the beans. When they are cooked, drain off the water. Melt an ounce of dripping in a frying-pan, lay in a large thinly sliced onion and fry it brown. Before the onion is quite dark enough add to it four ounces of raw bacon cut in cubes and fry it also. Add the onion, dripping, and bacon to the beans, with a careful seasoning to taste. Reheat it thoroughly, pile in a hot dish, dust with parsley chopped finely, and serve with mashed potatoes. If liked, a gill of stock or gravy can be added as well as bacon and onion.

"WARD SISTER" inquires: What is the best method of cleaning varnished sanitary papers in bathrooms, etc.?

Clean them in the following manner. Sweep them down lightly with a clean soft duster tied over a broom-head. Work from cornice to floor, and if very dusty change the duster frequently. Next dissolve two tablespoonfuls of powdered borax in four tablespoonfuls of boiling water. Add to this three pints of cold water. Sponge or wipe the paper over with this, letting the strokes overlap so as to avoid a streaky appearance. Dry with a soft clean non-fluffy cloth. If the walls are very dirty, warm, not hot, water may have to be used, slightly lathered with dissolved soap. The paper must then be rinsed with cold water to re-harden the varnish, as the warmth of the water tends to make it sticky. If time permits the paper can after drying be rubbed over with a clean chamois leather to remove all marks.

"LAUNDRY NOVICE" is anxious to know whether paraffin washing is to be recommended for the cleansing of very dirty coarse clothes, or will it injure material?

Paraffin is frequently used as a cleansing agent for very greasy and dirty coarse articles. A paraffin wash saves much rubbing, and if used for infected articles of clothing with a long boiling it destroys spores and germs. The following points will however need special attention. The clothes, after washing, must be very thoroughly rinsed in hot rinsing water and dried in the open air, otherwise the smell of paraffin lingers. If a greasy scum rises on the boiling water more shredded soap must be added, or this scum will boil into the clothes and discolour them. The clothes should be put in dry, not previously steeped and washed; this, of course, saves soap, time, and trouble. Lastly, special care is needed to clean the copper after a paraffin wash to remove all grease or smell.

"HASTINGS" inquires: How can slimy sponges be treated? Soda seems to have no effect.

Alkaline solutions are useless to remove slime from sponges. Put them in strong salt and water for several hours, changing the water if necessary. Then rinse them thoroughly in clean water; thread a loop of string through them and allow them to hang outside in the air until dry. If the sponges have been much neglected and the above remedy is insufficient, mix one sherry-glassful of muriatic acid with three pints of water and soak them in this mixture, then rinse and dry as directed above.

"A. L." writes: How can ironmould be removed from a quantity of linen? Salts of lemon was applied, but it caused holes.

The application of salts of lemon to remove ironmould from the linen was quite correct; that it caused holes to appear showed that the remedy was wrongly applied. Try this method: Dip the stain in boiling water and arrange the wet portion over a basin. Sprinkle the stain with salts of lemon, rub it in for a second or two, and then pour on to the mark quite boiling water. This is most easily done from the spout of a kettle. Next rinse in cold water to which has been added half a teaspoonful of bicarbonate of soda to each half pint of water. The alkali checks the action of the acid. Lastly, well wash the linen in the usual manner.

"HOUSEKEEPER" writes: What causes tables and wooden floors to become a bad colour although frequently and thoroughly scrubbed?

Frequent hard scrubbing does not ensure wood being a good colour. Probably some or all of the following errors are committed by the scrubbers. They neglect to brush up loose dirt on floors before wetting them. They use the same water for the entire process no matter how dirty it may become; this washes dirt into the wood, and is a very common fault. They put soda in the water; this soon darkens and discolours wood. They scrub across, not with the grain of the wood; thus the brushes do not penetrate into the grooves. They neglect thoroughly to rinse off the dirty soapy water. The flannel should be dipped into clean water before drying the wood.

"DISSATISFIED" wants to know if there is any better looking material for fixing at the back of stoves, sinks, and lavatory basins than galvanised zinc.

Instead of galvanised zinc for this purpose try the enamelled zinc "Emdeca." It is made in various patterns and colours to resemble tiles, and is pretty and sanitary, washable, and easily fixed. It costs about 9d. per square foot.

"NURSE ETTA" writes: I find my barley-water is frequently a little bitter, though I prepare it very carefully. Can you tell me how this defect can be remedied?

A slightly bitter flavour is characteristic of barley, but it can be almost if not entirely removed by blanching the barley. To do this, wash the barley under the cold-water tap, put it in a saucepan with sufficient cold water to cover it well, and bring it to boiling-point. Boil for five minutes, then strain off the water, rinse the barley again and use as desired.

"A VOICE FROM CUMBERLAND": Our kitchen sadly lacks modern improvements, and there is much difficulty in keeping patients' dinners hot. Can you suggest any simple method that will prevent the food becoming dry, etc.?

The best plan for keeping a number of dinners hot without the food drying is to adopt the "bain marie" system. A large shallow pan containing boiling water is placed on the stove, and in this the covered mess-tins, dishes, or saucepans can stand and the contents be kept just below boiling-point while the surrounding water actually boils. A local ironmonger should be able to make you a large strong tin or iron pan about four or five inches deep and of a length and breadth to fit your stove.

